# Influence of AGTR1 and ABCB1 Gene Polymorphism on the Curative Effect of Irbesartan

**DOI:** 10.1155/2022/4278675

**Published:** 2022-11-09

**Authors:** Qiao Wang, Lingsen You, Zeyu Li, Leiyi Zhang, Xueqi Li, Xuesong Yang

**Affiliations:** ^1^Department of Cardiovascular Medicine, The Fourth Affiliated Hospital of Harbin Medical University, Harbin 116000, China; ^2^Department of Cardiovascular Medicine, Dalian University Affiliated Xinhua Hospital, Dalian 116021, China; ^3^Division of Histology and Embryology, Joint Laboratory for Embryonic Development & Prenatal Medicine, Medical College, Jinan University, Guangzhou 510632, China; ^4^Department of Cardiovascular Medicine, The First Affiliated Hospital of Jiamusi Medical University, Jiamusi 114003, China

## Abstract

The interindividual heterogeneity in response to the antihypertensive effect of irbesartan has received considerable attention because of gene polymorphism. In this study, we investigated the new combinational influences of AGTR1 and ABCB1 gene polymorphism on the therapeutic effect of irbesartan among Chinese hypertensive patients. A total of 353 samples including 168 normal people and 185 hypertensive patients were adopted, and genotypes comprise ABCB1 (CC, CT, and TT) and AGTR1 (AA and AC) in this study. The results of multiple linear regression models showed that no statistically significant differences were observed in blood pressure change following irbesartan administration in each genotype from either ABCB1 (CC, CT, and TT) or AGTR1 (AA and AC). However, spline smoothing analysis demonstrated that the blood pressure therapeutic responses of irbesartan presented a noticeable difference among different ABCB1 genotypes when irbesartan doses reached over 300 ng/mL. Eventually, we assumed that the different drug responses of irbesartan among various AGTR1 genotypes were due to the diversity of the irbesartan-conjugated protein, which is responsible for crossing-coupled intracellular G-protein-coupled receptors (GPCRs).

## 1. Introduction

Essential hypertension (EH, also called primary hypertension) is defined as a rise in blood pressure (i.e., persistent systolic/diastolic blood pressure readings are 140/90 mmHg or more) of undetermined cause [1]. Hypertension could directly or indirectly cause the increased risk of cardiac, cerebral, and renal damages, thereby eventually being closely associated with augmented mortality and morbidity from coronary heart disease, congestive heart failure, end-stage renal failure, and stroke if it is not adequately controlled [2]. It is estimated that hypertension disturbs roughly more than one billion people around the world [3]. Therefore, it is very important that antihypertensive drugs are administered to lower blood pressure to prevent the complications of hypertension. Due to the differences in effects of antihypertensive drugs, some hypertensive patients have to take more than two drugs for blood pressure control [4]. We had to admit that there are still some problems in hypertension management and its concomitant risks in particular patient subgroups in spite of the effectiveness and safety of antihypertensive drugs [1, 2, 5].

In regard to hypertension treatment, there is not any therapy of elimination and eradication for hypertension. Therefore, for the earlier stages of hypertension, doctors adopt preventive strategies (i.e., controlling obesity, high fat/sodium dietary intake, smoking and alcohol abuse, and getting more exercise) to reduce the incidence of hypertension complications (i.e., heart attack, kidney damage, and stroke); for established hypertension, the combination strategy of pharmacotherapy and abovementioned lifestyle modification will be emphasized [3]. Irrespective of the availability of dozens of antihypertensive drugs at the moment, the hypertension treatment outcome has been far from ideal. Therefore, the direction for antihypertensive therapy in the near future should be put forward to improve blood pressure control with the available drugs through adequate drug and dose combinations, as well as discretely tailored gene polymorphism-directed therapies (e.g., gene therapy and vaccines) [3, 4].

As one of the first-line antihypertensive drugs, irbesartan is orally administered alone or with other antihypertensive drugs, in a dose of 150–300 mg/day, for the treatment of mild to moderate hypertension in clinics [6, 7]. The pharmacodynamics of irbesartan is that it suppresses the bioactivity of angiotensin II (Ang II) selectively and noncompetitively binds to AII receptor subtype 1 (AT_1_), which is deemed to regulate most of the physiological activities of AII [8]. Angiotensin receptor blockers (ARBs) manifested the effectiveness of lowering blood pressure compared with other kinds of antihypertensive drugs, such as ACE inhibitors (ACE-I), *β*-receptor blockers, calcium channel blockers (CCBs), and diuretics [9, 10]. The most common adverse events of irbesartan administration include headache, upper respiratory tract infection, muscle pain, dizziness, and exhaustion [6, 11]. Actually, for the treatment of hypertensive patients, it is still obscure what is a determinant factor in each individual patient's response to irbesartan treatment. Nevertheless, growing evidence suggests that gene polymorphism in drug-targeted receptors or metabolizers is related to distinct response to antihypertensive drugs like irbesartan [12], which implies that it is necessary to identify the correlation between gene polymorphism in particular patients and irbesartan therapeutic effects [12–15].

Moreover, the study of breeds using molecular techniques is very important and useful for identifying their characteristics [16–18]. The determination of genetic traits requires the proper performance of conservative superiorities that should be based on universal information on population structures, including genetic diversity and resources between breeds and individuals [19, 20]. Genetic diversity is an essential element for implementing genetic improvement, preserving diversity in populations, and adapting to variable environmental situations [21, 22]. On the other hand, determination of gene polymorphism is likewise important [23, 24] since it provides genotypes and their associations with different traits [18, 25, 26]. Of note, gene polymorphism can have a direct impact on drug interactions and therapies via suppressing or inducing some key enzyme activities and metabolizers, which probably influence the pharmacological effect of irbesartan [27]. In this study, we focused on the correlations between the therapeutic effect of irbesartan and the angiotensin II type 1 receptor (AGT1R) or ATP-binding cassette subfamily B member 1 (ABCB1) [28] gene polymorphism under the following considerations. First, irbesartan, as an AGT1R-specific antagonist, can specifically block the binding of Ang II to AGT1R (i.e., the final step of the renin-angiotensin system), thereby exerting its antihypertensive effect [29]. There seems to be substantial interindividual variations towards different responses of irbesartan with the same effective dose as previously reported [12, 30, 31]. As one of the ATP-binding cassette (ABC) genes, ABCB1 is presented in all kinds of tissues and cells and responsible for cellular homeostasis [32]. In particular, it encodes transporter proteins possessing multiple membrane-spanning domains for ATP-dependent translocation of substrates across the cell membrane [33, 34]. Obviously, ABCB1 appears to be functionally associated with the renin-angiotensin aldosterone system (RAAS), one of the main pathogeneses of hypertension.

## 2. Material and Methods

### 2.1. Patient Population and Selection

Patients with mild-to-moderate hypertension were enrolled from The First Affiliated Hospital of Jiamusi Medical University, Jiamusi, China, from June 2015 to July 2016 (Ethics number: IRB-AF/25–1.0). In total, 194 males and 159 females, which include 168 healthy controls and 185 patients, were recruited to participate in the study (Table 1). In brief, the inclusion criteria of healthy controls were as follows: age 18–77 years, systolic blood pressure (SBP) 90–130 mmHg, and diastolic blood pressure (DBP) 60–79 mmHg, while there were not any abnormal indexes in other routine tests, such as the blood biochemical test and routine urine test. Meanwhile, the inclusion criteria of hypertensive patients were as follows: systolic blood pressure (SBP) 140–179 mmHg or diastolic blood pressure (DBP) 90–109 mmHg, aged 18–77 years old, and without taking any antihypertensive medications within 4 weeks before the study. The patients with any of the following conditions were excluded: secondary hypertension, pregnancy, hypercalcemia, chronic cardiovascular disease, chronic cerebrovascular disease, chronic liver or renal diseases, or body mass index (BMI) greater than 33 kg/m^2^. Each subject underwent a detailed history and physical examination by an experienced internist. This study was approved by the Ethics Committee of Jiamusi Medical University. The purpose and procedures of the study were carefully explained to all participants, and written informed consent was obtained from all participants.

### 2.2. Measurement of Blood Pressure and the Survey of Blood Pressure-Related Variables

SBP and DBP were measured by well-trained nurses using a mercury-gravity manometer with appropriately sized cuffs after the patients rested in a seated position for at least half an hour without smoking or having tea or coffee. A questionnaire survey was conducted to obtain relevant baseline characteristics, including occupation, education background, alcohol intake/smoking history, age, gender, weight, height, and other demographic factors. Additionally, for the patients with diabetes mellitus, fasting plasma glucose levels at baseline and corresponding treatment were collected.

### 2.3. Irbesartan Administration and Blood Sample Collection

All patients were instructed to take a 150 mg tablet of irbesartan (manufactured by Sanofi-Synthelabo, France) once daily in the morning, on an empty stomach, for 2, 4, and 8 consecutive weeks. Irbesartan doses were administered at our study center for the first and the 2^nd^, 4^th^, and 8^th^ weeks; i.e., patients were asked to come to the study center every 7 days for the follow-up medication for next 7 days. During the follow-up, patients were requested to record the time they took medication and any side effects in a standardized diary. Patients were excluded from the study if they were unable to tolerate drug therapy or took any other medications during the follow-up. Blood were collected at the time point from predose on the first day and the specific time point before drug administration. Blood plasma was obtained from blood samples after centrifuging (5417R Centrifuge; Eppendorf AG, Hamburg, Germany) at 48°C and stored in a −80°C freezer before analysis.

### 2.4. High-Performance Liquid Chromatography (HPLC) Determination of Irbesartan in Human Plasma

Plasma irbesartan concentration was determined using HPLC with fluorescence detection as described in a previous study [35]. To ensure the precision and accuracy in this assay, quality control samples were prepared to contain five different irbesartan concentrations within the standard curve range and were demonstrated with the mean, SD, and coefficients of variation in the intraday and interday measurements.

### 2.5. DNA Extraction, Single Nucleotide Polymorphism (SNP) Selection, and Genotyping

Venous blood samples were collected from all participants. Genomic DNA was extracted using the QIAamp Blood Kit (Qiagen, USA) by standard techniques and stored at −20°C until genotyping. SNP in AGT1R and ABCB1 genes was genotyped using the TaqMan allelic discrimination method (Applied Biosystems, Foster City, California, USA). We selected SNPs with minor allele frequency (MAF) more than 0.1 in the population of China, and genotyped SNPs were evenly distributed across the AGT1R gene with an average distance of 15 kb SNPs.

### 2.6. Statistical Analysis

The SAS statistical program (Release 8.0; SAS Institute, Cary, North Carolina, USA) was used for data analysis. Descriptive statistical analyses were presented as percentages for categorical variables and means (SD) for continuous variables. The between-group data were compared using Student's *t*-test between two groups or one-way ANOVA among three groups for continuous variables and the *χ*2 test for categorical variables. A LOWESS smoothing curve was produced to illustrate a correlation between the BP baseline and the BMI. Blood pressure (BP) response was defined as the BP before treatment minus BP on 28^th^ day. The effect of the SNP on BP response to irbesartan was estimated by multiple linear regression models with adjustment for potential confounding variables. Genotypes, that is, AGTR1 (AA and AC) and ABCB1 (CC, CT, and TT), were included in a regression model as 0/1 dummy variables. We also examined the additive interaction effect between SNP and plasma irbesartan concentration on BP responses. A spline smoothing curve of BP therapeutic response in relation to plasma concentration of irbesartan was established through being stratified by ABCB1 genotypes.

## 3. Results

### 3.1. The Baseline Information of the Samples Employed in This Study

**Figure 1:**
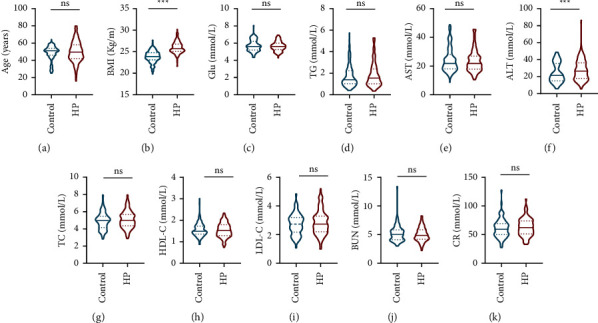
The relevant assessment for various factors that might be associated with the therapeutic effect of irbesartan on controlling blood pressure. (a)–(k) The comparisons of demographic characteristics including age (a), BMI (body mass index) (b), GLU (glucose) (c), TG (triglyceride) (d), AST (aspartate transaminase) (e), ALT (alanine transaminase) (f), TC (total cholesterol) (g), HDL-C (high-density lipoprotein cholesterol) (h), LDL-C (low-density lipoprotein cholesterol) (i), BUN (blood urea nitrogen) (j), and CR (creatinine) (k) between control and hypertensive patients (HPs).

For hypertensive patients, no responders were taking other antihypertensive drugs except for irbesartan during the study. For demographic information, there is a similar population of males and females, and they were both similarly aged at an average of roughly 48–50 years. Numerous biochemical and physiological indexes in human peripheral blood serum and urine were measured in both groups (Table 1), and the result showed that there were not statistically significant differences between the control and hypertension group except for BMI and ALT (Figure 1, Table 1).

**Figure 2:**
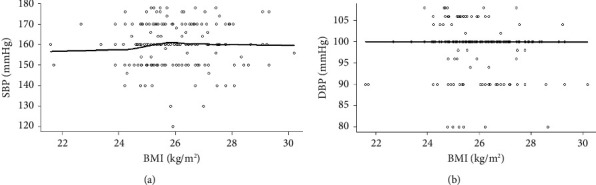
Assessing SBP/DBP baseline in the context of different BMIs. (a, b) The values of SBP (a) and DBP (b), respectively, in the context of different BMIs.

Furthermore, among the objects in this study, we did not observe any relevance between the BMI of the objects and SBP (systolic blood pressure) and DBP (diastolic blood pressure) (Figure 2).

### 3.2. Similar Distribution of Polymorphism of AGTR1 and ABCB1 in the Samples Employed in This Study

Genotype frequency distributions were determined among several groups of people employed in this study and showed that there were not statistically significant differences in several subtypes of either AGTR1 or ABCB1 (Table 2).

Similarly, no statistically significant differences in subtype distributions in AGTR1 or ABCB1 were witnessed among age, gender, BMI, and baseline SBP/DBP (Table 3).

### 3.3. Diversities of Hypertensive Effects among AGTR1 and ABCB1 Subtypes in Response to Irbesartan

First, we did not find statistically significant differences in the hypertensive effects of irbesartan of certain concentrations among the genotypes of either ABCB1 (CC, CT, and TT) or AGTR1 (AA and AC) (Table 4).

**Figure 3:**
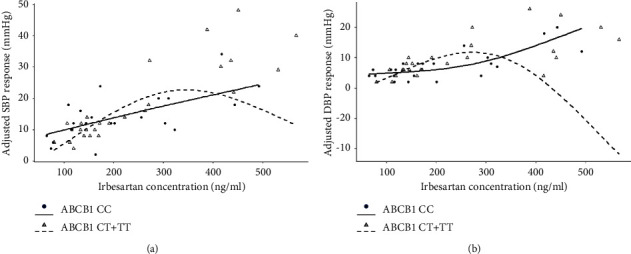
Assessing the therapeutic responses of SBP/DBP between two ABCB1 genotypes. (a)-(b) The spline smoothing plots of adjusted SBP response (a) and adjusted DBP response (b), respectively, in the context of different plasma irbesartan concentrations (ng/mL).

However, the hypertensive effects of higher concentrations of irbesartan became statistically different in the diverse genotypes of ABCB1 (i.e., ABCB1 CC and ABCB1 CT + TT), which could be observed on the spline smooth plots of blood pressure responses to irbesartan (Figure 3).

**Figure 4:**
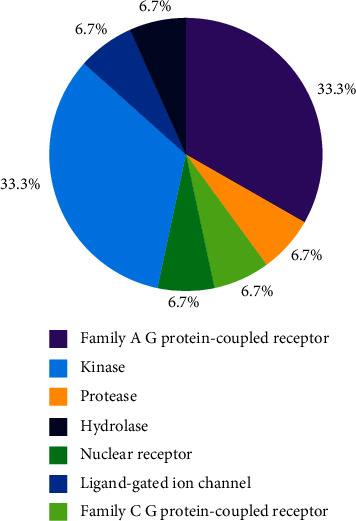
The components of binding protein of irbesartan.

In order to investigate the underlying mechanism of the aforementioned blood pressure responses to irbesartan, we converted the measurement data into categorical variables and then compared them with the analysis of variance. The result showed that there were statistically significant differences in the genotypes of AGTR1 despite not showing any significant differences in the ABCB1 genotype. Interestingly, we found that the maximum component in the binding site of irbesartan and GPCRs was the family A subtype G-protein-coupled receptor (Figure 4).

The pie chart shows the proportion of binding proteins of irbesartan, predicted by SwissTargetPrediction (http://www.swisstargetprediction.ch/).

## 4. Discussion

Firstly, we could eliminate the possibility that the blood pressure lowing effects of irbesartan are not derived from the patients' demographic characteristics, such as gene genotype distribution, and the baselines of SBP/DBP and BMI-SBP. It is because we found statistically significant differences in BMI and ALT between control and patients (Table 1, Figure 1) in the data of demographic characteristics, and there were no other statistically significant differences in the assessments of the aforementioned items (Figure 2, Tables 2 and 3). Unexpectedly, no statistically significant differences were observed in blood pressure change following irbesartan administration in each genotype from either ABCB1 (CC, CT, and TT) or AGTR1 (AA and AC) (Table 4), which seems contradictory with the previous study in the AGTR1 genotype reported by Jiang el at. [12]. However, when spline smoothing (i.e., a potent approach for evaluating functional relationships between the predictor and response) was employed to analyze the blood pressure therapeutic responses of irbesartan, we found a significant difference which was worth mentioning; that is, the significant hypertensive effect occurred among different ABCB1 genotypes when irbesartan doses reached over 300 ng/mL (Figure 3). When we converted the measurement data of SBP and DBP in response to irbesartan administration into categorical variables, the result from the chi-square analysis demonstrated a significant difference in therapeutic effect of irbesartan between AGTR1 genotypes despite the fact that no significant difference was discovered when their data were analyzed as measurement data format previously (Table 5).

Drug response is dependent on the combined effect of a variety of factors, such as patients' age, body state, concomitant therapy, and drug interactions. Among numerous factors, the sequence variants in patients' genes encoding drug-metabolizing enzymes or drug-targets are obviously not to be sniffed at and of great value to clinical medication. This is because the observed therapeutic effect is actually a mirror of biochemical and physiological adaptations between the drug and its target. Compared to other influencing factors, the genetic factor of drug response is generally stable throughout patients' lifetime [36]. So, in this study, why did a variety of irbesartan hypertensive effects occur in high-dose administration among different ABCB1 genotypes? To address this issue, we converted the measurement data of SBP and DBP in response to irbesartan administration into categorical variables and then carried out the chi-square analysis. After a series of the abovementioned processes, we were surprised to find that there was a significant difference in therapeutic effect of irbesartan between AGTR1 genotypes despite the fact that no significant difference was discovered when their data were analyzed as measurement data format previously (Table 5).

This further facilitates the mechanism investigation of aforementioned phenotype. During the pursuit process, we pleasantly found that the largest component in the binding site of irbesartan and GPCRs was the family A subtype G-protein-coupled receptor (Figure 4). As we know, GPCRs acts as the majority of membrane receptors, which can efficiently and selectively transmit chemical signals of ligand-receptor interactions to intracellular response elements [37], such as a hypertensive effect of antihypertensive drug is achieved through targeting on various loci in the renin-angiotensin system (RAS) [38]. Thus, we can reasonably assume that different drug responses of irbesartan among various AGTR1 genotypes were due to the diversity of the irbesartan-conjugated protein, which is responsible for crossing-coupled intracellular GPCRs.

The limitation of this prospective study mainly included the following two parts. First, the enrollment numbers of both hypertension patients and healthy controls were restricted because of objective condition. Second, we only chose to study the most important SNPs among AGT1R/ABCB1 genes rather than completely investigate many other SNPs, which might function in the anti-hypertension effect of irbesartan as well.

To sum up, our data demonstrated a significant interaction between plasma irbesartan concentration and AGTR1/ABCB1 in SBP/DBP responses, although their manifestations were different. This is to say that various hypotensive effects occur in the patients with different subtypes of AGTR1. Meanwhile, the patients with diverse subtypes of ABCB1 do not show significant difference in response to same plasma concentration of irbesartan. However, in the context of adjusting serum irbesartan concentration, we found that the patients with different ABCB1 subtypes illustrated a different trend of antihypertension, suggesting a role for AGTR1/ABCB1 gene variants in modulating individual responses to irbesartan antihypertensive therapy. There is no doubt that more precise and larger cohort studies are required to confirm our findings.

## Figures and Tables

**Table 1:** The sample demographic information in this study.

Variables	Control *n* = 168	Patient *n* = 185	*t*	*p*
Gender	93/75	101/84		
Age	48.37 ± 8.439	49.96 ± 12.18	1.410	0.1595
BMI	23.83 ± 1.361	25.82 ± 2.218	10.060	<0.0001
GLU	5.70 ± 0.66	5.60 ± 0.68	1.422	0.156
TC	4.92 ± 0.975	5.08 ± 0.996	1.566	0.118
HDL-C	1.55 ± 0.310	1.540 ± 0.329	0.311	0.756
LDL-C	2.716 ± 0.729	2.851 ± 0.827	1.621	0.106
TG	2.716 ± 0.729	1.86 ± 1.132	0.517	0.605
AST	24.26 ± 8.338	23.70 ± 8.00	0.642	0.521
ALT	24.25 ± 10.780	27.71 ± 11.94	2.848	0.005
BUN	5.150 ± 1.353	5.510 ± 1.26	0.589	0.557
CR	63.20 ± 16.950	63.13 ± 15.50	0.088	0.93

Note. BMI, body mass index; GLU, glucose; TC, total cholesterol; HDL-C, high-density lipoprotein cholesterol; LDL-C, low-density lipoprotein cholesterol; TG, triglyceride; AST, alanine aminotransferase; ALT, alanine aminotransferase; BUN, blood urea nitrogen; CR, creatinine.

**Table 2:** The genotype frequency distribution in patient, control, and published article groups showed no significant difference.

	*n*	Genotype frequency (%)			Allele frequency (%)		*χ * ^2^	*p*
AGTR1								
rs5186		AA	AC		A	C		
Patient	185	165 (89.2)	20 (10.8)		94.6	5.4		
Control	168	152 (90.5)	16 (9.5)		95.2	4.8	0.622	0.733

ABCB1								
rs1045642		CC	CT	TT	C	T		
Patient	185	58 (31.5)	87 (48.0)	40 (20.5)	54.9	45.1		
Control	168	54 (32.0)	85 (50.3)	30 (17.8)	56.8	43.2	1.797	0.407

**Table 3:** Each genotype patient's basic information illustrated no significant difference.

	Age	Gender	BMI	Baseline SBP	Baseline DBP
AGTR1					
AA	49.74 ± 12.15	86/79	25.57 ± 2.920	158.4 ± 10.05	98.92 ± 5.488
AC	45.22 ± 13.77	9/11	26.06 ± 1.267	160.9 ± 10.67	98.41 ± 6.164
P	0.1484	0.362	0.1361	0.1009	0.5518

ABCB1					
CC	48.86 ± 12.09	28/29	25.74 ± 1.295	159.6 ± 9.580	99.44 ± 5.151
CT	51.15 ± 12.02	46/43	26.11 ± 1.439	160.0 ± 10.82	98.58 ± 5.948
TT	48.85 ± 12.71	22/18	25.81 ± 1.215	158.7 ± 10.68	97.80 ± 6.354
P	0.4436	0.325	0.2259	0.8187	0.3859

**Table 4:** Each genotype patient's blood pressure change.

Genotype	*n*	ΔSBP	ΔDBP	ΔPP
ABCB1				
CC	57	16.35 ± 3.647	9.509 ± 3.323	6.842 ± 2.328
CT	89	16.31 ± 4.111	9.101 ± 2.680	7.213 ± 3.498
TT	39	15.95 ± 3.324	9.436 ± 2.712	6.513 ± 3.136
F		0.1541	0.3999	0.7416
P		0.8573	0.671	0.4778

AGTR1				
AA	165	16.25 ± 3.717	9.273 ± 2.914	6.982 ± 3.159
AC	20	16.20 ± 4.538	9.500 ± 2.743	6.700 ± 2.618
F		1.025	1.265	1.457
P		0.6966	0.6032	0.7021

**Table 5:** Effects of irbesartan in different genotype patients.

Genotype	*n*	Highly effective	Effective	Ineffective	Total effective	Total effective ratio (%)	*χ*2
ABCB1							
CC	57	14	26	17	40	70.18	*χ * ^2^ = 3.137, contingency coefficient = 0.129 *p* = 0.535
CT	89	19	38	32	57	64.04	
TT	39	10	21	8	31	79.49	

AGTR1							
AA	165	39	73	53	112	67.87	*χ * ^2^ = 8.638, contingency coefficient = 0.211 *p* = 0.013
AC	20	4	12	4	16	80.00	

## Data Availability

The data used to support the findings of this study are included within the article.

## References

[1] Carretero O. A., Oparil S. (2000). Essential hypertension: part I: definition and etiology. *Circulation*.

[2] Staessen J. A., Wang J., Bianchi G., Birkenhager W. H. (2003). Essential hypertension. *The Lancet*.

[3] Israili Z. H., Hernández-Hernández R., Valasco M. (2007). The future of antihypertensive treatment. *American Journal of Therapeutics*.

[4] Brown M. J., McInnes G. T., Papst C. C., Zhang J., MacDonald T. M. (2011). Aliskiren and the calcium channel blocker amlodipine combination as an initial treatment strategy for hypertension control (ACCELERATE): a randomised, parallel-group trial. *The Lancet*.

[5] Messerli F. H., Williams B., Ritz E. (2007). Essential hypertension. *The Lancet*.

[6] Bramlage P., Durand-Zaleski I., Desai N., Pirk O., Hacker C. (2009). The value of irbesartan in the management of hypertension. *Expert Opinion on Pharmacotherapy*.

[7] Kochar M., Guthrie R., Triscari J., Kassler Taub K., Reeves R. A. (1999). Matrix study of irbesartan with hydrochlorothiazide in mild-to-moderate hypertension. *American Journal of Hypertension*.

[8] Forni V., Wuerzner G., Pruijm M., Pruijm M. (2011). Long-term use and tolerability of irbesartan for control of hypertension. *Integrated Blood Pressure Control*.

[9] van den Meiracker A. H., Admiraal P. J., Janssen J. A. (1995). Hemodynamic and biochemical effects of the AT1 receptor antagonist irbesartan in hypertension. *Hypertension*.

[10] Kassler-Taub K., Littlejohn T., Elliott W., Ruddy T., Adler E. (1998). Comparative efficacy of two angiotensin II receptor antagonists, irbesartan and losartan, in mild-to-moderate hypertension. *American Journal of Hypertension*.

[11] Gillis J. C., Markham A. (1997). Irbesartan. A review of its pharmacodynamic and pharmacokinetic properties and therapeutic use in the management of hypertension. *Drugs*.

[12] Jiang S., Hsu Y. H., Venners S. A. (2011). Interactive effect of angiotensin II type 1 receptor (AGT1R) polymorphisms and plasma irbesartan concentration on antihypertensive therapeutic responses to irbesartan. *Journal of Hypertension*.

[13] Kurland L., Liljedahl U., Karlsson J. (2004). Angiotensinogen gene polymorphisms: relationship to blood pressure response to antihypertensive treatment: results from the Swedish Irbesartan Left Ventricular Hypertrophy Investigation vs. Atenolol (SILVHIA) trial. *American Journal of Hypertension*.

[14] Kurland L., Hallberg P., Melhus H. (2008). The relationship between the plasma concentration of irbesartan and the antihypertensive response is disclosed by an angiotensin II type 1 receptor polymorphism: results from the Swedish Irbesartan Left Ventricular Hypertrophy Investigation vs. Atenolol (SILVHIA) Trial. *American Journal of Hypertension*.

[15] Jiang S., Mao G., Zhang S. (2005). Individual and joint association of *α*-adrenergic receptor Arg347Cys polymorphism and plasma irbesartan concentration with blood pressure therapeutic response in Chinese hypertensive subjects. *Clinical Pharmacology & Therapeutics*.

[16] Mohammadi A., Nassiry M. R., Mosafer J., Mohammadabadi M. R., Sulimova G. E. (2009). Distribution of BoLA-DRB3 allelic frequencies and identification of a new allele in the Iranian cattle breed Sistani (*Bos indicus*). *Russian Journal of Genetics*.

[17] Mohammadabadi M. (2021). Tissue-specific mRNA expression profile of ESR2 gene in goat. *Agricultural Biotechnology Journal*.

[18] Gholamhoseinzadeh Gooki F., Mohammadabadi M., Asadi Fozi M. (2018). Polymorphism of the growth hormone gene and its effect on production and reproduction traits in goat. *Iranian Journal of Applied Animal Science*.

[19] Mohammadabadi M. R., Nikbakhti M., Mirzaee H. R. (2010). Genetic variability in three native Iranian chicken populations of the Khorasan province based on microsatellite markers. *Russian Journal of Genetics*.

[20] Mohammadabadi M. R., Jafari A. H. D., Bordbar F. (2017). Molecular analysis of CIB4 gene and protein in Kermani sheep. *Brazilian Journal of Medical and Biological Research*.

[21] Zamani P., Akhondi M., Mohammadabadi M. (2015). Associations of Inter-Simple Sequence Repeat loci with predicted breeding values of body weight in sheep. *Small Ruminant Research*.

[22] Mohammadabadi M., Bordbar F., Jensen J., Du M., Guo W. (2021). Key genes regulating skeletal muscle development and growth in farm animals. *Animals*.

[23] Mohammadabadi M., Soflaei M., Mostafavi H., Honarmand M. (2011). Using PCR for early diagnosis of bovine leukemia virus infection in some native cattle. *Genetics and Molecular Research*.

[24] Gooki F. G., Mohammadabadi M., Fozi M. A., Soflaei M. (2018). Association of biometric traits with growth hormone gene diversity in Raini Cashmere goats. *Walailak Journal of Science and Technology*.

[25] Nassiry M. R., Eftekhar Shahroodi F., Mosafer J. (2005). Analysis and frequency of bovine lymphocyte antigen (BoLA-DRB3) alleles in Iranian Holstein cattle. *Russian Journal of Genetics*.

[26] Norouzy A., Nassiry M. R., Eftekhari Shahrody F., Javadmanesh A., Mohammad Abadi M. R., Sulimova G. E. (2005). Identification of bovine leucocyte adhesion deficiency (BLAD) carriers in Holstein and Brown Swiss AI bulls in Iran. *Russian Journal of Genetics*.

[27] Schelleman H., Stricker B. H. C., de Boer A. (2004). Drug-gene interactions between genetic polymorphisms and antihypertensive therapy. *Drugs*.

[28] Kathawala R. J., Wang Y. J., Shukla S. (2015). ATP-binding cassette subfamily B member 1 (ABCB1) and subfamily C member 10 (ABCC10) are not primary resistance factors for cabazitaxel. *Chinese Journal of Cancer*.

[29] Goodfriend T. L., Elliott M. E., Catt K. J. (1996). Angiotensin receptors and their antagonists. *New England Journal of Medicine*.

[30] Pool J. L., Guthrie R. M., Littlejohn T. W. (1998). Dose-related antihypertensive effects of irbesartan in patients with mild-to-moderate hypertension. *American Journal of Hypertension*.

[31] Reeves R. A., Lin C. S., Kassler-Taub K., Pouleur H. (1998). Dose-related efficacy of irbesartan for hypertension: an integrated analysis. *Hypertension*.

[32] Hodges L. M., Markova S. M., Chinn L. W. (2011). Very important pharmacogene summary: ABCB1 (MDR1, P-glycoprotein). *Pharmacogenetics and Genomics*.

[33] Pei Q., Yang L., Tan H. (2017). Effects of genetic variants in UGT1A1, SLCO1B3, ABCB1, ABCC2, ABCG2, ORM1 on PK/PD of telmisartan in Chinese patients with mild to moderate essential hypertension. *International Journal of Clinical Pharmacology & Therapeutics*.

[34] Jones P. M., George A. M. (2004). The ABC transporter structure and mechanism: perspectives on recent research. *Cellular and Molecular Life Sciences*.

[35] Bae S. K., Kim M. J., Shim E. J. (2009). HPLC determination of irbesartan in human plasma: its application to pharmacokinetic studies. *Biomedical Chromatography*.

[36] Padmanabhan S. (2014). *Handbook of Pharmacogenomics and Stratified Medicine*.

[37] Hill S. J. (2006). G-protein-coupled receptors: past, present and future. *British Journal of Pharmacology*.

[38] Felder R. A., Sanada H., Xu J. (2002). G protein-coupled receptor kinase 4 gene variants in human essential hypertension. *Proceedings of the National Academy of Sciences*.

